# Transarterial Treatment of Direct Carotid Cavernous Fistulas with the Assistance of Undetachable Balloons

**DOI:** 10.1155/2013/152076

**Published:** 2013-05-16

**Authors:** Ning Xu, Yubo Wang, Qi Luo, Honglei Wang

**Affiliations:** Department of Neurosurgery, First Hospital of Jilin University, Changchun 130021, China

## Abstract

Directed carotid cavernous fistula means high blood flow shunts between the internal carotid artery and the cavernous sinus. Obstructing the abnormal shunt between the internal carotid artery and the cavernous sinus while preserving the internal carotid artery is the key role in fistula treatment. Transarterial balloon embolization is currently the gold standard treatment for most of the carotid cavernous fistulas. But there are still some technical difficulties in the use of detachable balloon to treat carotid cavernous fistulas. Here, we describe undetachable balloon-assisted technique in the embolization of three patients who got complete immediate occlusion of the shunt and preserved the internal carotid artery at the same time.

## 1. Introduction

Directed carotid cavernous fistula (CCF) means high blood flow shunts between the internal carotid artery (ICA) and the cavernous sinus (CS). Trauma is the chief reason of this kind of disease. Obstructing the abnormal shunt between the ICA and the CS while preserving the ICA is the key role in CCF treatment. Detachable balloon technique is an effective way in CCF treatment. But it does not always work. In this paper, we report our experiences in embolization of CCF using detachable balloon with the assistance of undetachable balloon.

## 2. Case Presentation

Sixteen patients suffering from CCF were treated with detachable balloons in our department from January 2009 to December 2011. Three of the 16 patients were treated with the assistance of undetachable balloons. All the patients were demonstrated by angiography. We tried to occlude the CCFs with detachable balloons via the arterial approach. An 8F guiding catheter was placed in the cervical part of the parent ICA. A detachable balloon (gold valve balloon, Nycomed, Paris, France) was mounted at the tip of the balloon catheter (Balt, France). And then the balloon was advanced to the ICA through the 8F guiding catheter. The balloon was inflated to occlude the opening of the fistula after passing through the orifice. An assistant undetachable balloon (HyperGlide, ev3, USA) was employed at the following circumstances. The detachable balloon could not pass through the orifice of the fistula or the inflated detachable balloon retracted to the ICA.

At the first circumstance, an undetachable balloon was introduced to the distal part of the ICA (distal part of the orifice of the CCF). The ICA was temporarily obstructed after inflating the undetachable balloon. The detachable balloon was successfully placed into the cavernous sinus because the orifice was the mainly drainage way of ICA at that time. Sometimes, the detachable balloon still could not pass through the orifice even if we did as aftermentioned. The undetachable balloon should be placed just at the orifice of the CCF. Inflating the undetachable balloon and then pushing the partially inflated detachable balloon into the cavernous sinus.

At the second circumstance, the undetachable balloon was placed at the orifice of the CCF, overlying the detachable balloon. The detachable balloon at the fistula was inflated to a proper size to occlude the orifice of the CCF. The detachable balloon couldn't protrude to the ICA under the support of the undetachable balloon. The angiogram after deflating the undetachable balloon confirmed the disappearance of the CCF and preserved ICA. The undetachable balloon should be inflated when detaching the detachable balloon to prevent its migration.

**Figure 1:**
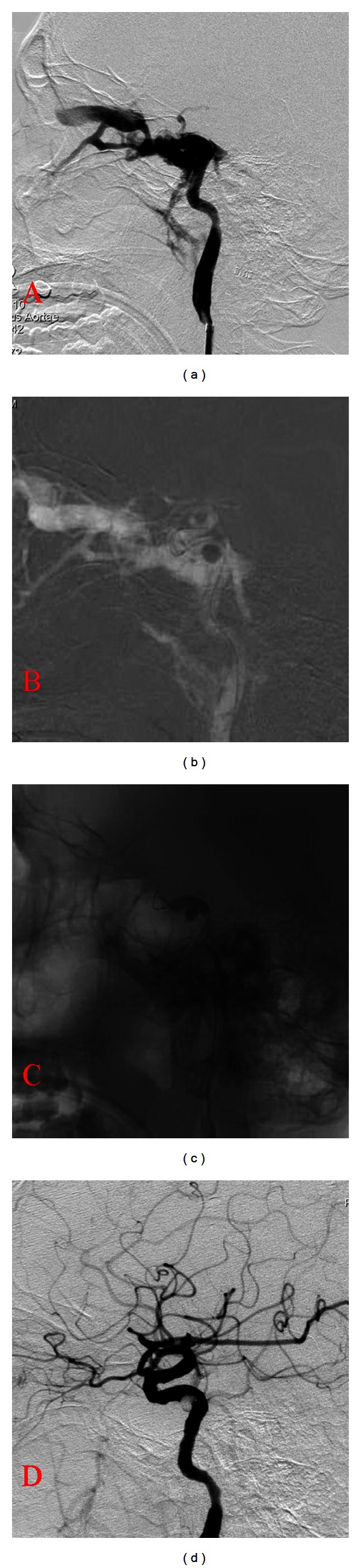
A 39-year-old male suffered from a right CCF after a history of head injury due to a traffic accident. (a) Lateral projection of right ICA angiogram shows a direct CCF. (b) The ICA was temporarily obstructed after inflating the undetachable balloon. The detachable balloon was successfully placed into the cavernous sinus because the orifice was the main drainage way of ICA at that time. (c) The undetachable balloon was placed at the orifice of the CCF, overlying the detachable balloon. The detachable balloon at the fistular was inflated to a proper size to occlude the orifice of the CCF. (d) Lateral projection of right ICA shows the disappearance of CCF and the preserved ICA.

Three of the 16 patients were treated with the assistance of undetachable balloon and got perfect outcomes (see Figure 1). All patients showed no recurrence of the symptoms at 12–18 months' followup. And repeated angiographies were therefore not performed. One of the 16 patients had obstructed ICA because of the huge orifice of the CCF.

## 3. Discussion

CCFs represent abnormal connections between the internal carotid artery (ICA) and external carotid arteries (ECAs) as well as the cavernous sinus (CS) or its dura. They are divided into indirect and direct categories [1]. Direct or type A fistulas involve direct connections between the ICA and CS via a hole in the cavernous segment ICA, which is the most common type. They could be caused by trauma, rupture of a cavernous carotid aneurysms, or a tear in the wall of a congenitally weak cavernous ICA secondary to collagen vascular disease. Many indirect type fistulas will close spontaneously but much fewer for the direct type. Direct CCFs always cause significant symptoms because of their high blood flow [2, 3]. Treatment of direct CCFs has progressed from surgical ligation to surgical trapping to endovascular techniques. Endovascular therapies are advantageous as they allow for occlusion of the fistula whilst preserving flow in the parent carotid artery. It is agreed that endovascular embolization of the fistula with detachable balloons or thrombogenic coil-based occlusion is safe and effective. As reported [4–6], coils or covered stents are also chosen to treat direct traumatic CCFs. Transarterial balloon embolization is currently the gold standard treatment for most of the CCFs [2, 3], and it is always the first choice for CCF treatment. But there are still some technical difficulties in the use of detachable balloon to treat CCFs. Undetachable balloon-assisted technique is a method to increase the success rate.

There are two key things that should be mentioned in the detachable balloon technique. The first is that the detachable balloon must be placed inside the cavernous sinus, brought to the orifice of the fistula, and inflated to a size that would stay at the orifice of the fistula. The second is that the detachable balloon should not protrude to the ICA to influence the blood flow of the ICA after inflation. The detachable balloons are flow directed. It is difficult to place the detachable balloon inside the cavernous sinus if the orifice of the CCF is not on the direction of the blood flow of the ICA or the orifice of the fistula is really small [7]. In this situation, an undetachable balloon is useful. In one of the 3 cases, we placed an undetachable balloon catheter (HyperGlide, ev3, USA) at the distal part of the ICA (distal part of the orifice of the CCF). The ICA was temporarily obstructed after inflating the undetachable balloon. The main drainage way of the ICA was the CCF. Then, the partially inflated detachable balloon floated into the cavernous sinus through the orifice of the fistula. Sometimes, we can place the undetachable balloon catheter at the orifice of the CCF. The undetachable balloon is as a bracing balloon to help pushing the detachable balloon pass through the fistula to the CS. The orifice of the fistula should be smaller than the inflated balloon so that the balloon could stay in the CS. Occasionally, when the width of the opening of the fistula is large enough, inflation of the detachable balloon may push itself back to the ICA or partially protrude to the ICA. This may lead to obstruction of the ICA. The detachable balloon inside the CS can be inflated to a size larger than the diameter of the opening of the fistula with the support of the inflated undetachable balloon at the orifice. Because it couldn't protrude to the ICA, the detachable balloon could be inflated to adapt the shape of the CS and remain inside the CS after removal of the undetachable balloon in the ICA. Another help of the undetachable balloon is to keep the stabilization of the detachable balloon when detaching. As we all know, the detachment of the balloon is by pulling back the catheter. Sometimes the pulling back of the catheter might cause migration of the detachable balloon. An inflated undetachable balloon at the orifice could prevent the occurrence of migration. The undetachable balloon assistant technique cannot work when the orifice of the fistula is too large or the CS is too small to accommodate the inflated balloon. At this time, other techniques should be chosen. Or the parent ICA should be occluded if there was good collateral circulation.

Teng and colleagues reported double-balloon technique to embolize CCF in 2000 [8]. The two balloons were all detachable balloons. There were some potential risks in using two detachable balloons. The detachable balloon has a potential trend to inflate to a ball. Overinflation of the balloon in the ICA may cause enlargement of fistula or rupture of the ICA. And because of the interaction of the two inflated balloons, they may be detached prematurely, which may result in occlusion of the ICA. In our cases, undetachable balloon is chosen as bracing balloon in ICA. It is specially designed to fix the shape of ICA and provides stable support to the undetachable balloon without damage to ICA and fistula. Premature detachment of the bracing balloon does not exist because it is undetachable. Tsai and colleagues introduced their experience in embolization of CCF with double-balloon techniques [9]. Their bracing balloon is undetachable balloon. Comparing to specially designed intracranial balloon, it still brings more stimuli to ICA and the fistula.

In conclusion, undetachable balloon-assisted detachable balloon embolization technique is very useful in the treatment of CCF. It significantly increases the accomplishment rate of CCF treatment. It is helpful in detachable balloon placing, inflation, and detachment.
